# Poor phenotype-genotype association in a large series of patients with Type III Bartter syndrome

**DOI:** 10.1371/journal.pone.0173581

**Published:** 2017-03-13

**Authors:** Alejandro García Castaño, Gustavo Pérez de Nanclares, Leire Madariaga, Mireia Aguirre, Álvaro Madrid, Sara Chocrón, Inmaculada Nadal, Mercedes Navarro, Elena Lucas, Julia Fijo, Mar Espino, Zilac Espitaletta, Víctor García Nieto, David Barajas de Frutos, Reyner Loza, Guillem Pintos, Luis Castaño, RenalTube Group, Gema Ariceta

**Affiliations:** 1 BioCruces Health Research Institute, Ciberer, Cruces University Hospital, Bizkaia, Spain; 2 Pediatric Nephrology, Cruces University Hospital, Bizkaia, Spain; 3 Department of Pediatrics, School of Medicine and Odontology, University of Basque Country UPV/EHU, Bizkaia, Spain; 4 Pediatric Nephrology, Vall d’Hebron University Hospital, Universitat Autonoma, Barcelona, Spain; 5 Pediatric Nephrology, Virgen del Camino Hospital, Pamplona, Spain; 6 Pediatric Nephrology, La Paz University Hospital, Madrid, Spain; 7 Pediatrics, Manises Hospital, Valencia, Spain; 8 Pediatric Nephrology, Virgen del Rocío Hospital, Sevilla, Spain; 9 Pediatric Nephrology, Fundación Alcorcón University Hospital, Madrid, Spain; 10 San Ignacio University Hospital, Bogotá, Colombia; 11 Pediatric Nephrology, Nuestra Señora de Candelaria University Hospital, Tenerife, Canarias, Spain; 12 Pediatric Nephrology, Virgen de las Nieves Hospital, Granada, Spain; 13 Nephrology Unit, Cayetano Heredia University, Cayetano Heredia Hospital, Lima, Peru; 14 Germans Trias i Pujol University Hospital, Badalona, Spain; 15 Centro de Investigación Biomédica en Red de Diabetes y Enfermedades Metabólicas Asociadas (CIBERDEM), Instituto de Salud Carlos III, Madrid, Spain; 16 Pediatric Nephrology, Asturias Central University Hospital, Oviedo, Asturias, Spain; Huashan Hospital Fudan University, CHINA

## Abstract

**Introduction:**

Type III Bartter syndrome (BS) is an autosomal recessive renal tubule disorder caused by loss-of-function mutations in the *CLCNKB* gene, which encodes the chloride channel protein ClC-Kb. In this study, we carried out a complete clinical and genetic characterization in a cohort of 30 patients, one of the largest series described. By comparing with other published populations, and considering that 80% of our patients presented the p.Ala204Thr Spanish founder mutation presumably associated with a common phenotype, we aimed to test the hypothesis that allelic differences could explain the wide phenotypic variability observed in patients with type III BS.

**Methods:**

Clinical data were retrieved from the referral centers. The exon regions and flanking intronic sequences of the *CLCNKB* gene were screened for mutations by polymerase chain reaction (PCR) followed by direct Sanger sequencing. Presence of gross deletions or duplications in the region was checked for by MLPA and QMPSF analyses.

**Results:**

Polyuria, polydipsia and dehydration were the main common symptoms. Metabolic alkalosis and hypokalemia of renal origin were detected in all patients at diagnosis. Calciuria levels were variable: hypercalciuria was detected in 31% of patients, while 23% had hypocalciuria. Nephrocalcinosis was diagnosed in 20% of the cohort. Two novel *CLCNKB* mutations were identified: a small homozygous deletion (c.753delG) in one patient and a small deletion (c.1026delC) in another. The latter was present in compound heterozygosis with the already previously described p.Glu442Gly mutation. No phenotypic association was obtained regarding the genotype.

**Conclusion:**

A poor correlation was found between a specific type of mutation in the *CLCNKB* gene and type III BS phenotype. Importantly, two *CLCNKB* mutations not previously described were found in our cohort.

## Introduction

Type III Bartter syndrome (BS), OMIM#607364, is an autosomal recessive disorder of salt reabsorption in the thick ascending limb (TAL) of Henle’s loop, characterized by hypokalemia, hypochloremic metabolic alkalosis, hyperreninemia, hyperaldosteronism with normal or low blood pressure and renal salt loss [1, 2]. This syndrome is caused by mutations in the *CLCNKB* gene (OMIM*602023), which encodes the chloride channel protein ClC-Kb [2–4]. This channel protein is required to ensure Cl^-^ exit on the basolateral cellular side. Thus, loss-of-function mutations in the *CLCNKB* gene reduce Cl^-^ exit and Na-K-Cl reabsorption by modifying the transepithelial voltage gradient and causing salt loss in urine [5].

Accurate clinical diagnosis of type III BS is complex as patients exhibit a wide phenotypic variability, which may hinder differentiation from other tubulopathies. Thus, mutations in the *CLCNKB* gene may cause overlapping phenotypes with other types of BS, such as type I BS (OMIM #601678) or antenatal BS type II (OMIM #241200) or with Gitelman syndrome (GS) (OMIM #263800) [6, 7].

There are few reports of large series of patients with genetically-confirmed type III BS. Our cohort of 30 patients is one of the largest to date. The aim of this study was to undertake a clinical and genetic characterization of this cohort and, taking advantage of the large number (80%) who presented the Spanish c.610G>A; p.Ala204Thr founder mutation [2, 8], evaluate the genotype-phenotype correlation between those patients and others with different mutations in the *CLCNKB* gene. Therefore, the presence of this founder mutation provides a unique opportunity to compare the phenotype associated with the p.Ala204Thr mutation with other published cohorts.

## Materials and methods

### Ethics statement

The study was approved by the Ethics Committee for Clinical Research (CEIC) of Cruces University Hospital. Patients and their relatives or guardians provided their written informed consent to participate in this study. Minors over 12 years of age also provided their consent.

### Population

A cohort of 30 patients (18 females) of whom 26 (87%) were Spanish was analyzed. The remaining four patients were from Colombia, Peru and two of African origin. Patient blood samples were referred through the Pediatric Nephrology Division at Cruces University Hospital (n = 13), the Renaltube network (n = 11) (www.renaltube.com), or from other hospitals in Spain (n = 6) and analyzed at the Molecular Genetic Laboratory at Cruces University Hospital.

### DNA analysis

Total DNA was extracted from EDTA-preserved peripheral blood leukocytes by the QIAamp^®^ DNA Blood Mini Kit (QIAGEN) method. The exon regions, promoter (described by SwitchGear Genomics) and flanking intronic sequences of the *CLCNKB* gene (Ensembl identifiers: gene ENSG00000184908; transcript, ENST00000375679) were screened for mutations by polymerase chain reaction (PCR) followed by direct sequencing (primer sequences and the strategy used to specifically amplify the exons of the *CLCNKB* gene were published previously [9]).

In order to detect large deletions or duplications in the *CLCNKB* region, a commercially-available MLPA (Multiplex Ligation Dependent Probe Amplification) kit, SALSA MLPA probemix P266-B1 (MRC Holland, Amsterdam, The Netherlands) was used. Quantitative Multiplex PCR of Short Fluorescent Fragments (QMPSF) assays were run to check for uncovered regions in the MLPA kit. QMPSF primers and conditions are available on request.

The pathogenic effect of DNA variants was assessed using prediction pathogenic software: Mutation t@sting (www.mutationtaster.org), SIFT (www.sift.jcvi.org), PolyPhen-2 (www.genetics.bwh.harvard.edu) MutPred (www.mutpred.mutdb.org) and SNPs&GO (www.snps-and-go.biocomp.unibo.it) (Table 1).

**Table 1:** Description of the *CLCNKB* mutations observed in our cohort and their *in silico* pathogenicity prediction.

Nucleotide level	Protein level	Exon	N	Polyphen2 *	SIFT ^†^	SNPs&GO ^‡^	MutPred ^§^	Mutation Taster ^||^
c.508G>A	p.Val170Met	6	1	probably damaging 0.99	damaging 0	disease-causing 1	deleterious mutation 0.66	disease-causing 0.99
c.610G>A	p.Ala204Thr	7	24	probably damaging 0.97	damaging 0	disease-causing 1	deleterious mutation 0.88	disease-causing 0.36
c.629C>T	p.Ala210Val	7	1	probably damaging 0.99	damaging 0	Neutral 1	deleterious mutation 0.95	disease-causing 0.99
**c.753delG**	**p.(Leu252fs)**	8	1	-	-	-	-	disease-causing 1
**c.1026delC**	p.(Ser343Alafs*6)	11	1	-	-	-	-	disease-causing 1
c.1192_1203del12	p.Ile398_Thr401del	12	1	-	-	-	-	disease-causing 0.90
c.1325A>G	p.Glu442Gly	14	5	probably damaging 1	damaging 0	disease-causing 3	deleterious mutation 0.71	disease-causing 0.99
c.1756+1G>A	p.?	16	1	-	-	-	-	-
c.1783C>T	p.Arg595*	17	1	-	-	-	-	disease-causing 1
c.(?_-1)_(*1_?)del	p.0	1–20	5	-	-	-	-	-

N: number of patients with the mutation;

*Score [range: benign 0- probably damaging 1];

^†^ Score (< 0.05 damaging, > 0.05 tolerable);

^‡^ Confidence [range: 0–10];

^**§**^ Probability (> 0.5 actionable hypotheses, > 0.75 confident hypotheses);

^**||**^ Probability [range: 0–1]; Mutations marked in bold have not been reported to date.

A diagnostic algorithm based on previous experiences was used: first, detection of the p.Ala204Thr Spanish founder mutation [2, 8]; second, detection of large deletions or duplications by MLPA and QMPSF; and third, sequencing of the rest of the coding and flanking regions of the whole *CLCNKB* gene [9].

## Results

### Patients’ medical reports

Clinical presentation was similar to that described in patients with type III BS, who manifest prenatal onset less frequently and whose calciuria is less pronounced compared with type I and II BS (Table 2) [2, 10–14].

**Table 2:** Clinical characteristics of the 30 patients in our cohort (expressed as number or mean ± SD).

	All patients (n = 30)	Patients with homozygous p.Ala204Thr mutation (n = 16)	*Student’s t-test / Mann-Whitney U*
Prenatal onset (polyhydramnios)	9/24	7/15	NS
Age at diagnosis (years)	1 [0.7–2.8]*	1.9 [0.7–2.5]*	
Gestational age (weeks)	39.6 ± 1.9	39.5 ± 2	
Gender	18 (female) / 12 (male)	9 (female) / 7 (male)	
Weight (SDS)	-2.6 [-3.8; -1.6]*	-3.2 [-3.9; -2.4]*	
Height (SDS)	-1.9 [-2.3; -1.0]*	-1.9 [-3; -1.4]*	
pH	7.49 ± 0.09	7.47 ± 0.09	
HCO_3_^-^ (mEq/L)	31.4 ± 7.4	29.2 ± 5	
P. Na (mEq/L)	134.1 ± 5.3	135.2 ± 4.4	
P. K (mEq/L)	2.4 ± 0.6	2.4 ± 0.7	
P. Cl (mEq/L)	89.6 ± 11.5	90.4 ± 7.2	
P. Creatinine (mg/dl)	0.4 [0.3–0.5]*	0.4 [0.3–0.5]*	
P. Mg (mg/dl)	2.1 ± 0.5	2.3 ± 0.6	
P. Ca (mg/dl)	10 ± 1.2	9.7 ± 1.4	
Renin (ng/ml/h)	40 [20–69]*	57 [20–80]*	
P. Aldosterone (pg/ml)	577 [271–1348]*	1113[624–1620]*	
FE Na%	0.5 [0.2–1.2]*	0.9 [0.2–1.3]*	
FE K%	31 [21–42]*	34 [19–50]*	
FE Cl%	1.2 [0.5–2.2]*	1.7 [0.4–2.3]*	
U Ca/Cr (mg/mg)	0.3 [0.1–0.5]*	0.3 [0.2–0.4]*	
TTKG	12.5 ± 3.1	12.2 ± 3.1	
U. Ca (mg/kg/d)	6.2 ± 5.4	9.2 ± 5.1	

Abbreviations: SDS, standard deviation score in comparison with an age- and sex-matched reference population; P, plasmatic; HCO3-, bicarbonate; FE, fractional excretion; TTKG, transtubular potassium gradient; U, urinary; Ca/Cr, calcium/creatinine ratio. NS, not statistically-significant.

Notes:

* Median [P_25_-P_75_]

Disease appeared early in life in 26 of 30 patients (87%) with a median of 1 year of age at diagnosis [P_25_ = 0.7, P_75_ = 2.8]. Four patients (13%) recalled a history of premature delivery and hydramnios was recorded in 9 out of 24 (37%). Remarkably, two patients were diagnosed in adulthood, at 17 and 25 years of age, despite being symptomatic.

Hypokalemia, the leading symptom, was observed in all patients and was associated with metabolic alkalosis and overactivity of the renin-angiotensin-aldosterone system (RAAS). However, the occurrence of hyponatremia and/or hypochloremia was variable, as was the amount of urinary calcium excretion.

The main symptoms were polyuria, polydipsia, recurrent vomiting, constipation, salt craving, dehydration, hypotonia and failure to thrive. Growth retardation and poor weight gain were almost universal (96%). Laboratory findings were consistent with metabolic alkalosis and hypokalemia of renal origin, commonly associated with hypochloremia and hyponatremia due to urine salt wasting or inappropriate urinary salt content. Increased plasma renin activity and aldosterone levels with normal blood pressure were the rule, and 6 patients had hypomagnesemia (S1 Table).

Characteristically, calciuria, as observed in other series published (S1 Table), was variable: hypercalciuria was detected in 31% and hypocalciuria in 23%. Nephrocalcinosis was diagnosed in 20% of patients (S1 Table). Interestingly, one patient had renal cysts in the absence of nephrocalcinosis.

### Analysis of mutation in the *CLCNKB* gene

Genetic diagnosis of this cohort had been partially described previously [9]. In summary, we detected ten different mutations in the *CLCNKB* gene (Table 1), with high a prevalence of the p.Ala204Thr Spanish founder mutation (24 patients from 21 families, 80%) [2, 8]: 16 patients (66.7%) from 16 families were homozygous and 8 (33.3%), from 5 families, compound heterozygous. Excluding those of non-Spanish origin, the founder mutation was detected in 92% of patients. Another two mutations were recurrent within the cohort (5 patients, 16.7%): the p.Glu442Gly mutation (exon 14) [9] (present in 3 siblings) and the entire gene deletion (c.(?_-1)_(*1_?)del) [2] (in 2 siblings).

Remarkably, two novel *CLCNKB* mutations were identified: one patient presented a small homozygous deletion in exon 8 (c.753delG; p.(Leu252fs)), while a second had another small deletion in exon 11 (c.1026delC; p.(Ser343Alafs*6)). In this latter case, the novel mutation was present in compound heterozygosis with p.Glu442Gly. Both novel mutations were considered disease-causative since these changes in the *CLCNKB* gene disrupt the reading frame and presumably lead to a truncated protein lacking the—COOH-terminus, thus hypothetically generating a faulty ClC-Kb channel unable to perform its function (Table 1).

### Correlations between *CLCNKB* mutations and clinical presentation

As mentioned previously, the p.Ala204Thr mutation was present in homozygosity in 16 patients; therefore, we were able to examine the phenotype of a large group of patients with the same alteration and ascertain whether there is any variability in the manifestation of the disease when all patients have the same alteration.

Regarding clinical symptoms and biochemical values within this group, a wide phenotype variability was observed as previously reported in patients with type III BS. Thus, prenatal symptoms were recorded in 7 patients (44%), while two recalled a history of premature delivery and hydramnios during pregnancy. All patients but one were diagnosed in infancy, with a median age of 1.9 years at diagnosis [P_25_ = 0.7, P_75_ = 2.5]. Polydipsia, polyuria, growth retardation and poor weight gain were common symptoms present during the early months of life. Furthermore, the occurrence of hypochloremia and hyponatremia was also variable: 11 patients (69%) presented hypochloremia (although the remaining 5 had chloride levels at the lower normal range limit) and 9 patients (56%) presented hyponatremia. Moreover, other biochemical features such as serum creatinine and magnesium levels and chloride and sodium fractional excretions were also variable (S2 Table).

Regarding the renal calcium excretion, the main clinical characteristic of study patients was the variability in urinary calcium excretion. Seven patients (44%) presented hypercalciuria while one had hypocalciuria (6%). The remaining six (50%) had normal urine calcium levels. Laboratory tests showed a median of 0.3 mg/mg (P_25_-P_75_: 0.2–0.4) for the urine calcium/creatinine clearance ratio in those patients.

Furthermore, some patients with truncating mutations (SOR0080: p.[Arg595*];[Arg595*], SOR0081: p.[(Ser343Alafs*6)];[Glu442Gly]) presented symptoms resembling those of other patients with the p.[Ala204Thr];[Ala204Thr] genotype (S2 Table). Therefore, a genotype-phenotype correlation could not be observed in our cohort.

### Long-term prognosis

With the aim of providing additional information on the long-term phenotype of type III BS patients, we examined the long-term clinical characteristics and renal outcome in 15 patients with *CLCNKB* mutations who were followed up for several years at our own institution (Table 3).

**Table 3:** Long-term prognosis and clinical characteristics of 15 patients with CLCNKB mutations after 17 [14–2,2]* years of follow-up (expressed as number or mean ± SD).

-	At diagnosis	Last follow-up	*t*	At diagnosis	Last follow-up	*t*	At diagnosis	Last follow-up	*t*	At diagnosis	Last follow-up	*t*	At diagnosis	Last follow-up	At diagnosis	Last follow-up
(n = 15)	*Height (SDS)*			*P*. *K (mEq/L)*			*U*. *Ca (mg/kg/d)*			*U Ca/Cr (mg/mg)*			*Proteinuria*		*Nephrocalcinosis*	
SOR0003	-0.11	-0.01		1.4	2.6		11	3.2		0.43	0.07		+	-	-	-
SOR0005	-0.97	-0.06		2	2.2		9.7	3.7		0.5	0.31		-	-	+	+
SOR0008	-3.16	-1		2	2.7		5.9	2.8		0.31	0.13		-	-	+	-
SOR0009	-3.48	0.1		1.7	3		9.2	1.3		0.33	NA		+	-	-	-
SOR0011	-2.33	-0.34		2	2.6		2.2	NA		NA	NA		-	-	-	-
SOR0023	-3.66	-1.3		3.3	2.7		NA	1		0.73	0.1		-	-	-	-
SOR0024	1.83	0.6		2.6	2.6		NA	3.1		NA	0.13		-	-	-	-
SOR0024	-1.2	0.8		1.8	2.3		0.5	0.08		NA	0.04		-	-	-	-
SOR0025	-1.96	-0.2		1.4	3.2		NA	1.7		0.23	0.18		-	-	-	-
SOR0026	-1.92	-0.5		3.3	2.5		2.2	7.8		0.2	0.26		-	-	-	-
SOR0045	-2.3	-0.6		2.3	4		NA	4		NA	0.2		-	+	-	-
SOR0050	-5.35	-0.95		2.2	3.2		NA	1.3		0.03	0.04		-	-	-	-
SOR0054	-0.27	NA		2.3	3.3		NA	NA		0	0.2		-	+	-	-
SOR0054	0.58	NA		2.2	2.8		NA	NA		0.53	0.02		-	-	-	-
SOR0054	-2.06	NA		2.9	2.8		NA	NA		0	0.05		-	+	-	-
	-1.76 ± 1.85	-0.29 ± 0.63	*P*<0.05	2.2 ± 0.6	2.8 ± 0.5	*P*<0.05	5.8 ± 4.2	2.7 ± 2.1	NS	0.3 ± 0.2	0.1 ± 0.1	*P*<0.05	2/15	3/15	2/15	1/15

Abbreviations: NA, not available; SDS, standard deviation score in comparison with an age- and sex-matched reference population; P, plasmatic; U, urinary; NS, not statistically-significant.

Notes:

* Median [P_25_-P_75_]

All received treatment with oral potassium supplements and indomethacin (± spironolactone). Duration of follow-up since clinical diagnosis was 18.6 ± 9.4 years. As a group, in comparison with baseline, the stature of study patients become normal. Their glomerular filtration rate was mostly preserved, with proteinuria in 3 patients. However, in 2 cases, mild chronic kidney disease was observed at diagnosis and at the end of the follow-up period despite the absence of nephrocalcinosis. Although treated, hypokalemia persisted almost universally but was less pronounced than at diagnosis, whereas the amount of urinary calcium excretion dropped to normal limits in most cases: at the last follow-up, one patient showed a urinary calcium increase of 2.2 to 7.3 mg/kg/day, while urinary calcium excretion decreased in the remaining patients. Only 1 of 15 presented nephrocalcinosis in the long term, but two patients had nephrolithiasis (Table 3).

## Discussion

Our results, and the comparison with previously-published cohorts (S1 Table), confirm the phenotypic variability in type III BS patients and the poor correlation with specific *CLCNKB* gene mutations in the majority of our patients, thereby highlighting the need for confirmatory molecular diagnosis of the disease.

Further, in our cohort as a whole, a phenotypic distinction could not be proven between patients with the homozygous p.Ala204Thr mutation and the remaining patients. Although differences in the clinical picture among individual patients with other *CLCNKB* gene mutations were minor, we cannot be sure they were related to specific mutations.

Regarding phenotype, and despite the genetic heterogeneity among described type III BS cohorts (S1 Table), the finding of hypokalemia, metabolic alkalosis and increased levels of circulating renin and aldosterone was almost universal in all published series [15] including our cohort. In fact, hypokalemia was observed in all patients of the cohort, and it has been reported that type III BS courses with the most severe and persistent hypokalemia among BS subtypes [16]. These metabolic data explain the characteristic clinical features of BS (S1 Table). Although Simon and Konrad did not specify the detection of other electrolyte abnormalities [2, 10], we also observed hyponatremia and hypochloremia in our cohort, as already reported by Bettinelli and Lee [11, 13].

It is commonly accepted that patients with confirmed type III BS do not usually have a history of polyhydramnios or premature birth due to less severe renal salt wasting and less intense polyuria [16]. However, as reported by Konrad and others [6, 16], we observed that ≈30% of our patients exhibited a pattern of antenatal BS. Thus, the presence or absence of prenatal symptoms also supports the variability of type III BS presentation.

This clinical variability in type III BS also applies for the Gitelman Syndrome (GS) phenotype, characterized by low calcium in urine [6, 16]. In contrast to other types of BS, type III usually manifests with lower levels of urinary calcium and more pronounced hypokalemia, as detected in this study. Indeed, 6 patients (23%) were found to have hypocalciuria. Nozu also reported hypocalciuria in all patients with mutations in the *CLCNKB* gene. In that study, 7 of 9 patients carried the same mutation (c.1830G>A) [12].

On the other hand, types I and II BS are associated with hypercalciuria due to the inability to reabsorb the calcium filtered coupled with Na-K-2Cl cotransporter activity at the TAL [17]. Despite general acceptance that type III BS is associated with low or normal calciuria, 8 patients in our cohort presented hypercalciuria (31%), as also reported by Simon in 11 of 17 patients [2]. Concurring with the idea of a less frequent presence of nephrocalcinosis in type III BS, we observed nephrocalcinosis in 20% of patients in our series and the development of nephrolithiasis in four cases, a fact also mentioned [18].

It has been hypothesized that different mutations in the *CLCNKB* gene could determine the presence or absence of hypocalciuria or hypercalciuria [12]. Our finding of variable calcium excretion in urine supports the difficulty of certifying the diagnosis of type III BS without performing a molecular analysis. Further, the calciuria level cannot help to establish a differential diagnosis with GS or other Bartter subtypes, and over time there was a trend towards normalization of the amount of calcium in urine. Renal cyst formation was another infrequent characteristic observed in some patients diagnosed with type III BS [19]. Renal cysts were detected in one patient, a finding that has been attributed to chronic hypokalemia and early nephrocalcinosis [19].

As mentioned previously, little information is available on long-term follow-up in type III BS. The long-term clinical and renal outcome of 13 patients with the same genotype (p.[Ala204Thr];[Ala204Thr]) was examined; in these patients, the evolution was favorable with preserved renal function and without proteinuria. Our study provides additional information on the long-term natural history of Bartter III in patients diagnosed early in life and treated correctly.

With respect to genotype, molecular defects were quite similar in many populations, although slight differences did occur. Thus, homozygous deletion encompassing the whole *CLCNKB* gene represented the most common molecular finding in type III BS in heterogeneous cohorts [2, 10, 20], being observed in up to half of the reported cases, even in patients of different ethnicity. Those authors described a more severe phenotype and hypercalciuria in patients with homozygous gross deletions, which is supported by our findings: the few patients with complete gene deletion in our cohort (such as patient SOR0097, with a homozygous entire gene deletion; or patient SOR0090, with the homozygous p.(Leu252fs)), also had more severe disease, with greater urinary salt wasting and hypercalciuria.

By contrast, several studies in more homogeneous type III BS populations [11–13, 21, 22] showed that whole gene deletions, though observed, tended to be present in compound heterozygosity with point mutations. In particular, Bettinelli found 8 different point mutations and 4 gross deletions in a homogeneous population of mostly Italian patients (the complete deletion of the *CLCNKB* gene was only found in 23% of patients) [11]. Furthermore, in two large series of Japanese and Korean patients, the c.1830G>A mutation was the most prevalent in both countries, with the complete deletion of the *CLCNKB* gene being the second more frequently observed mutation [12, 13]. This is similar to what was observed in our Spanish population, where 80% of the studied patients presented the p.Ala204Thr mutation in the *CLCNKB* gene and whole gene deletion was less frequent. In these last three countries, founder mutations were the cause of the majority of type III Bartter cases.

In summary, this study shows the wide spectrum of clinical and biochemical characteristics observed both in patients sharing the p.Ala204Thr mutation and in those with other mutations in the *CLCNKB* gene, even within members of the same family, a phenomenon already reported [7], thus providing additional evidence of the need for a genetic diagnosis in clinical practice. Moreover, that finding ruled out the value of some parameters such as the level of urinary calcium excretion or the absence/presence of nephrocalcinosis to orient the differential diagnosis between distinct Bartter syndrome subtypes and even with Gitelman syndrome as suggested in the past [12]. Many studies attempted to explain this heterogeneity as variations in the expression of ClC-Kb channels, in the function of the channels involved in Cl^-^ transport in Henle’s loop or distal convoluted tubule, or in the variation of the expression or function of ClC-Ka channels. Finally, the different observed polymorphisms could affect the activity of the protein implicated in Cl^-^ transport [23–24]. We found more than 40 different SNP in our study (data not shown). Considering the minor allele frequency (MAF) established by the 1000 Genomes Project, these SNP, with the exception of p.Pro683Leu, are relatively frequent in the European population and were previously described as non-pathogenic polymorphisms. However, the p.Pro683Leu polymorphism is a rare variant in the general population, and although it was described as a non-pathogenic polymorphism, a negative effect on the protein was predicted by prediction pathogenic software. We observed this heterozygous polymorphism in three patients (SOR0054-1, SOR0057 and SOR0081), but did not see a more severe phenotype in these patients.

In conclusion, our results confirm the type III BS phenotype in patients of our cohort and, as described in the literature, that a poor correlation exists between a specific type of mutation in the *CLCNKB* gene and a particular phenotype or clinical severity. This clinical heterogeneity hinders diagnosis of the entity and renders it almost impossible to differentiate GS from type III BS, and even from other types of BS, thereby highlighting the need for a confirmatory genetic diagnosis, which requires orienting molecular analysis. Importantly, the complete genetic study in our cohort revealed two not previously reported *CLCNKB* mutations: the c.753delG; p.(Leu252fs) and c.1026delC; p.(Ser343Alafs*6) small deletions in exons 8 and 11, respectively.

## Supporting information

S1 Table Comparison of clinical and biochemical characteristics of different cohorts of patients with confirmed type III BS. (DOC)

S2 Table Clinical and biological characteristics of type III BS patients. (DOC)
