# Supplementary material for: Poor phenotype-genotype association in a large series of patients with Type III Bartter syndrome
Source: PLoS One. 2017 Mar 13;12(3):e0173581. doi: 10.1371/journal.pone.0173581 (PMC5348002; doi:10.1371/journal.pone.0173581)
Supplement: S1 Table — (DOC) [file pone.0173581.s001.doc]

Poor phenotype-genotype association in a large series of patients with type III Bartter syndrome.

Alejandro García Castaño^1^, Gustavo Pérez de Nanclares^1^, Leire Madariaga^2, 3^, Mireia Aguirre^2^, Álvaro Madrid^4^, Sara Chocrón^4^, Inmaculada Nadal^5^, Mercedes Navarro^6^, Elena Lucas^7^, Julia Fijo^8^, Mar Espino^9^, Zilac Espitaletta^10^, Víctor García Nieto^11^, David Barajas de Frutos^12^, Reyner Loza^13^, Guillem Pintos^14^ Luis Castaño^1, 3, 15^, RenalTube Group^1, 4, 11, 16^, Gema Ariceta^4^.

1 BioCruces Institute, Ciberer, Cruces University Hospital, Bizkaia, Spain; 2 Pediatric Nephrology, Cruces University Hospital, Bizkaia, Spain; 3 Department of Pediatrics, School of Medicine and Odontology, University of Basque Country UPV/EHU, Bizkaia, Spain; 4 Pediatric Nephrology, Vall d’Hebron University Hospital, Universitat Autonoma, Barcelona, Spain; 5 Pediatric Nephrology, Virgen del Camino Hospital, Pamplona, Spain; 6 Pediatric Nephrology, La Paz University Hospital, Madrid, Spain; 7 Pediatrics, Manises Hospital, Valencia, Spain; 8 Pediatric Nephrology, Virgen del Rocío Hospital, Sevilla, Spain; 9 Pediatric Nephrology, Fundación Alcorcón University Hospital, Madrid, Spain; 10 San Ignacio University Hospital, Bogotá, Colombia; 11 Pediatric Nephrology, Nuestra Señora de Candelaria University Hospital, Tenerife, Canarias, Spain; 12 Pediatric Nephrology, Virgen de las Nieves Hospital, Granada, Spain; 13 Nephrology Unit, Cayetano Heredia University, Cayetano Heredia Hospital, Lima, Peru; 14 Germans Trias i Pujol University Hospital, Badalona, Spain; 15 Centro de Investigación Biomédica en Red de Diabetes y Enfermedades Metabólicas Asociadas (CIBERDEM), Instituto de Salud Carlos III, Madrid, Spain; 16 Pediatric Nephrology, Asturias Central University Hospital, Oviedo, Asturias, Spain.

**Corresponding author:** gariceta@vhebron.net

**S1 Table. Comparison of clinical and biochemical characteristics of different cohorts of patients with confirmed type III BS.**

|  | **This study (n=30)** | | **Simon *et al.* (n=17) [2]** | | **Konrad *et al.* (n=36) [10]** | | **Peters *et al.* (n=20)** **[14]** | | **Bettinelli *et al.* (n=13) [11]** | | **Lee *et al.* ( n=23) [13]** | |
| --- | --- | --- | --- | --- | --- | --- | --- | --- | --- | --- | --- | --- |
|  | **n** | **%** | **n** | **%** | **n** | **%** | **n** | **%** | **n** | **%** | **n** | **%** |
| **Polyhydramnios** | 9/24 | 37 | NA | NA | 8/25 | 32 | 5/20 | 25 | 2/13 | 15 | NA | ±22 |
| **Diagnosis ≤ 1 year** | 16/30 | 53 | 12/17 | 70 | 22/34 | 65 | 11/20 | 55 | 10/13 | 77 | 20/23 | 87 |
| **Hypokalemia*** | 30/30 | 100 | 17/17 | 100 | 36/36 | 100 | 13/20 | 65 | 13/13 | 100 | 23/23 | 100 |
| **Hyponatremia*** | 17/29 | 59 | NA | NA | NA | NA | NA | NA | 7/13 | 54 | NA | NA |
| **Hypochloremia*** | 21/28 | 75 | NA | NA | NA | NA | 5/20 | 25 | 13/13 | 100 | 23/23 | 100 |
| **Metabolic alkalosis*** | 27/29 | 93 | 17/17 | 100 | NA | 100 | 4/20 | 20 | 13/13 | 100 | 23/23 | 100 |
| **Magnesemia*** | 6/26 (Low) | 23 | 17/17 (Normal) | 100 | 9/23 (Low) | 39 | NA (Low) | ±50 | 3/13 (Low) | 23 | 5/23 (Low) | 22 |
| **Hyperreninemia*** | 20/20 | 100 | 6/6 | 100 | NA | 100 | NA | NA | 13/13 | 100 | 23/23 | 100 |
| **Hyperaldosteronism*** | 19/22 | 86 | 6/6 | 100 | NA | 100 | NA | NA | 13/13 | 100 | 23/23 | 100 |
| **Calciuria*** | 6/26 (Low) 8/26 (High) | 23 31 | 11/17 ( High ) | 65 | 2/25 (Low) / 7/25 (High) | 8/ 28 | 10/20 (High) | 50 | 2/13 (Low) / 1/13 (High) | 15/ 8 | 13/23 (High) | 56 |
| **Nephrocalcinosis** | 6/30 | 20 | 0/17 | 0 | 4/25 | 16 | 2/20 | 10 | 0/13 | 0 | 4/23 | 17 |

Abbreviations: n, number of patients; NA, not available. *According to our laboratory reference values.
