# Supplementary material for: Poor phenotype-genotype association in a large series of patients with Type III Bartter syndrome
Source: PLoS One. 2017 Mar 13;12(3):e0173581. doi: 10.1371/journal.pone.0173581 (PMC5348002; doi:10.1371/journal.pone.0173581)
Supplement: S2 Table — (DOC) [file pone.0173581.s002.doc]

Poor phenotype-genotype association in a large series of patients with type III Bartter syndrome.

Alejandro García Castaño^1^, Gustavo Pérez de Nanclares^1^, Leire Madariaga^2, 3^, Mireia Aguirre^2^, Álvaro Madrid^4^, Sara Chocrón^4^, Inmaculada Nadal^5^, Mercedes Navarro^6^, Elena Lucas^7^, Julia Fijo^8^, Mar Espino^9^, Zilac Espitaletta^10^, Víctor García Nieto^11^, David Barajas de Frutos^12^, Reyner Loza^13^, Guillem Pintos^14^ Luis Castaño^1, 3, 15^, RenalTube Group^1, 4, 11, 16^, Gema Ariceta^4^.

1 BioCruces Institute, Ciberer, Cruces University Hospital, Bizkaia, Spain; 2 Pediatric Nephrology, Cruces University Hospital, Bizkaia, Spain; 3 Department of Pediatrics, School of Medicine and Odontology, University of Basque Country UPV/EHU, Bizkaia, Spain; 4 Pediatric Nephrology, Vall d’Hebron University Hospital, Universitat Autonoma, Barcelona, Spain; 5 Pediatric Nephrology, Virgen del Camino Hospital, Pamplona, Spain; 6 Pediatric Nephrology, La Paz University Hospital, Madrid, Spain; 7 Pediatrics, Manises Hospital, Valencia, Spain; 8 Pediatric Nephrology, Virgen del Rocío Hospital, Sevilla, Spain; 9 Pediatric Nephrology, Fundación Alcorcón University Hospital, Madrid, Spain; 10 San Ignacio University Hospital, Bogotá, Colombia; 11 Pediatric Nephrology, Nuestra Señora de Candelaria University Hospital, Tenerife, Canarias, Spain; 12 Pediatric Nephrology, Virgen de las Nieves Hospital, Granada, Spain; 13 Nephrology Unit, Cayetano Heredia University, Cayetano Heredia Hospital, Lima, Peru; 14 Germans Trias i Pujol University Hospital, Badalona, Spain; 15 Centro de Investigación Biomédica en Red de Diabetes y Enfermedades Metabólicas Asociadas (CIBERDEM), Instituto de Salud Carlos III, Madrid, Spain; 16 Pediatric Nephrology, Asturias Central University Hospital, Oviedo, Asturias, Spain.

**Corresponding author:** gariceta@vhebron.net

**S2 Table. Clinical and biological characteristics of type III BS patients**

| ***Patient*** | *Sex* | *Age at Dx (years)* | *Weight (SDS)* | *Height (SDS)* | *Gest Age (weeks)* | *PH* | *pH blood* | *P. HCO_3_^-^*(mEq/L) | *P. Na* (mEq/L) | *P. K* (mEq/L) | *P. Cl* (mEq/L) | *P. Creat* (mg/dl) | *P. Mg* (mg/dl) | *P. Ca* (mg/dl) | *P. renin activity* (ng/ml/h) | *P. aldost* (pg/ml) | *FE Na (%)* | *FE K (%)* | *FE Cl (%)* | *TTKG* | *U Ca/Cr (mg/mg)* | *U Ca (mg/kg/d)* | *NC* |
| --- | --- | --- | --- | --- | --- | --- | --- | --- | --- | --- | --- | --- | --- | --- | --- | --- | --- | --- | --- | --- | --- | --- | --- |
| **p.[Ala204Thr];[Ala204Thr]** | | | | | | | | | | | | | | | | | | | | | | | |
| *SOR0003 | F | 6.8 | -1.29 | -0.11 | 36 | + | 7.53 | 35 | 134 | 1.4 | 100 | 0.67 | 0.9 | NA | 20.9 | 945 | 1.2 | 49.9 | 2.9 | NA | 0.43 | 11 | - |
| *SOR0005 | F | 3 | -1.63 | -0.97 | NA | + | 7.5 | 27.9 | 130 | 2 | 85 | 0.5 | 2.2 | 9.4 | 60.5 | 484 | 2.8 | 50.5 | 4 | 10.8 | 0.5 | 9.7 | + |
| *SOR0008 | F | 2 | -4.88 | -3.16 | 40 | - | 7.47 | 28 | 134 | 2 | 88 | 0.68 | 1.5 | 9.7 | NA | NA | 1.5 | 62.3 | 2.12 | NA | 0.31 | 5.9 | + |
| *SOR0009 | F | 2 | -4.50 | -3.48 | 36 | + | 7.31 | 20 | 143 | 1.7 | 100 | 0.75 | NA | 10 | 13.6 | NA | 1.33 | 76.8 | 2.37 | NA | 0.33 | 9.2 | - |
| *SOR0023 | M | 1.8 | -3.18 | -3.66 | NA | - | 7.47 | 24.3 | 139 | 3.3 | 85 | 0.36 | 1.9 | 5.2 | 53.4 | 267 | 1.2 | 27.5 | 2.09 | NA | 0.73 | NA | - |
| *SOR0025 | F | 2.3 | -3.73 | -1.96 | 40 | - | 7.57 | 26.7 | 132 | 2.3 | 95 | 0.46 | 1.5 | 10.7 | 215 | 5610 | 1.2 | 34.2 | 2 | NA | 0.23 | NA | - |
| *SOR0026 | F | 0.7 | -3.73 | -1.92 | 40 | + | 7.42 | 36.9 | 135 | 3.3 | 90 | 0.2 | 2.5 | 10.6 | 13.6 | 1654 | 0.14 | 7.2 | 0.19 | 10.8 | 0.2 | 2.2 | - |
| *SOR0039 | F | 1.4 | -4.05 | -2.34 | 41 | - | 7.49 | 28 | 138 | 1.6 | 93 | 0.3 | 2.8 | 10.1 | 86.6 | 1281 | 2.3 | 54 | 3.5 | NA | 0.2 | NA | - |
| *SOR0045 | M | 0.7 | 2.02 | -2.3 | NA | + | 7.48 | 39.9 | 133 | 2.3 | 79 | 0.45 | 2.3 | NA | NA | 670 | 0.18 | 13.4 | 0.31 | NA | NA | NA | - |
| *SOR0047 | M | 0.7 | -3.46 | -1.90 | NA | - | 7.65 | 31 | 137 | 2.5 | 83 | 0.28 | 3.1 | 10.8 | NA | 760 | 0.15 | 17.7 | 0.28 | 12.1 | 0.15 | NA | - |
| *SOR0048 | F | 2 | -2.33 | -1.23 | 42 | + | 7.38 | 27 | 126 | 2.7 | 82 | 0.16 | 2.9 | 10.5 | 77.4 | 282 | 0.6 | 14.2 | 0.6 | 8.2 | 0.23 | NA | - |
| *SOR0050 | M | 0.7 | -5.26 | -5.35 | 41 | - | 7.5 | 31.4 | 140 | 2.2 | 99 | 0.3 | 2.4 | 8.9 | NA | NA | 0.25 | 20.8 | NA | 16.9 | 0.03 | NA | - |
| *SOR0051 | M | 25 | NA | NA | NA | NA | 7.34 | 23.6 | 134 | 2.3 | 98 | 2.68 | 2.6 | 9.6 | NA | NA | NA | NA | NA | NA | NA | NA | + |
| *SOR0062 | M | 3 | -2.67 | -1.89 | 40 | - | NA | 30.7 | 130 | 1.9 | 89 | 0.33 | 2.4 | 9.7 | 80.4 | 1608 | 0.9 | 42 | 1.4 | NA | 1.1 | 17.5 | + |
| *SOR0073 | F | 0.7 | -3.19 | -0.98 | 40 | - | 7.47 | 29.9 | 139 | 3.3 | 97 | 0.4 | 2.2 | 10.4 | 8.2 | 1370 | 0.001 | 25 | 0.3 | 14.5 | 0.38 | NA | - |
| SOR0084 | M | 0.5 | -2 | -0.82 | 38 | + | 7.46 | 27.1 | 137 | 3.7 | 83 | 0.4 | 2.4 | 10.8 | 40 | 1900 | 0.31 | 34.8 | 0.62 | NA | 0.12 | NA | - |
| **MEAN** |  | **3.1** | **-2.93** | **-2.23** | **39.5** | **7/15** | **7.47** | **29.2** | **135** | **2.4** | **90** | **0.56** | **2.2** | **9.7** | **60.9** | **1403** | **0.94** | **35.35** | **1.62** | **12.2** | **0.35** | **9.2** | **4/16** |
| **SD** |  | **6.1** | **1.79** | **1.34** | **2** | **-** | **0.09** | **5** | **4.4** | **0.7** | **7** | **0.59** | **0.6** | **1.4** | **58.6** | **1434** | **0.83** | **20.17** | **1.28** | **3.1** | **0.28** | **5.1** | **-** |
| **p.[Ala204Thr];[0]** | | | | | | | | | | | | | | | | | | | | | | | |
| *SOR0011 | F | 11 | NA | -2.33 | NA | - | 7.45 | 29.4 | 138 | 2 | 93 | 0.5 | 1.7 | 10 | 31.1 | 1015 | 0.81 | 31.4 | 1.11 | NA | NA | 2.2 | - |
| *SOR0024-1 | F | 17 | 1.00 | 1.83 | 40 | - | NA | 33 | 132 | 2.6 | 134 | 0.4 | 1.3 | 9.8 | 195 | 114 | NA | NA | NA | NA | NA | NA | - |
| *SOR0024-2 | F | 0.9 | -4.97 | -1.2 | NA | NA | NA | NA | NA | 1.8 | NA | 0.5 | NA | NA | NA | NA | NA | 12 | NA | NA | NA | 0.5 | - |
| **MEAN** |  | **9.6** | **-1.98** | **-0.6** | **40** | **0/2** | **7.45** | **31.2** | **135** | **2.1** | **113** | **0.47** | **1.5** | **9.9** | **113.1** | **564** | **0.81** | **21.7** | **1.11** | **-** | **-** | **1.4** | **0/3** |
| **SD** |  | **8.1** | **4.23** | **2.2** | **-** | **-** | **-** | **2.5** | **4.2** | **0.4** | **29** | **0.06** | **0.3** | **0.1** | **115.9** | **637** | **-** | **13.7** | **-** | **-** | **-** | **1.2** | **-** |
| **p.[Ala204Thr];[Glu442Gly]** | | | | | | | | | | | | | | | | | | | | | | | |
| *SOR0054-1 | M | 0.3 | -2.36 | -0.27 | NA | - | 7.51 | 32.6 | 131 | 2.3 | 80 | 0.51 | 2.2 | 10.6 | NA | NA | 0.29 | 42.1 | 0.6 | NA | 0 | NA | - |
| *SOR0054-2 | F | 0.5 | -0.77 | 0.58 | NA | - | 7.4 | 26.5 | 145 | 2.2 | 81 | 0.5 | NA | NA | 60.6 | 220 | 0.22 | 30.9 | NA | NA | 0.53 | NA | - |
| *SOR0054-3 | F | 0.3 | -2.23 | -2.06 | NA | - | 7.46 | 28.4 | 134 | 2.9 | 86 | 0.66 | 2.2 | NA | 21 | 185 | 0.47 | 25 | 1.19 | NA | 0 | NA | - |
| *SOR0057 | F | 0.7 | -6.19 | -3.49 | 36 | + | 7.62 | 35.3 | 134 | 2.3 | 87 | 0.29 | 2.8 | 10.7 | 39 | 473 | 0.14 | 24.3 | 0.49 | 16.6 | 0.43 | NA | + |
| **MEAN** |  | **0.5** | **-2.89** | **-1.31** | **36** | **1/4** | **7.5** | **30.7** | **136** | **2.4** | **83** | **0.49** | **2.4** | **10.7** | **40.2** | **293** | **0.28** | **30.6** | **0.76** | **16.6** | **0.24** | **-** | **1/4** |
| **SD** |  | **0.2** | **2.32** | **1.82** | **-** | **-** | **0.09** | **4** | **6.2** | **0.3** | **3.5** | **0.15** | **0.4** | **0.07** | **19.8** | **157** | **0.14** | **8.2** | **0.38** | **-** | **0.28** | **-** | **-** |
| **p.[Ala204Thr];[Val170Met]** | | | | | | | | | | | | | | | | | | | | | | | |
| *SOR0064 | M | 0.8 | -2.57 | -1.69 | 41 | + | 7.55 | 43 | 134 | 3.1 | 81 | 0.33 | 2.2 | 9.4 | 19.9 | 1584 | 0.17 | 8.2 | 0.23 | 8 | 0.54 | 1.5 | - |

| ***Patient*** | *Sex* | *Age at Dx (years)* | *Weight (SDS)* | *Height (SDS)* | *Gest Age (weeks)* | *PH* | *pH blood* | *P. HCO_3_^-^*(mEq/L) | *P. Na* (mEq/L) | *P. K* (mEq/L) | *P. Cl* (mEq/L) | *P. Creat* (mg/dl) | *P. Mg* (mg/dl) | *P. Ca* (mg/dl) | *P. renin activity* (ng/ml/h) | *P. aldost* (pg/ml) | *FE Na (%)* | *FE K (%)* | *FE Cl (%)* | *TTKG* | *U Ca/Cr (mg/mg)* | *U Ca (mg/kg/d)* | *NC* |
| --- | --- | --- | --- | --- | --- | --- | --- | --- | --- | --- | --- | --- | --- | --- | --- | --- | --- | --- | --- | --- | --- | --- | --- |
| **p.[Ile398_Thr401del];[0]** | | | | | | | | | | | | | | | | | | | | | | | |
| *SOR0063 | F | 0.4 | -2.91 | -1.05 | 41 | - | 7.59 | 33.5 | 133 | 2.7 | 94 | 0.33 | 2.3 | 11.8 | 30 | 384 | 0.26 | 35.2 | 1.3 | NA | 0.12 | NA | - |
| **p.[Ala210Val];[?]** | | | | | | | | | | | | | | | | | | | | | | | |
| *SOR0076 | F | 1 | 1.53 | -1.61 | NA | - | 7.46 | 28 | 138 | 2.7 | NA | 0.6 | NA | NA | 50 | 480 | NA | NA | NA | NA | NA | NA | - |
| **p.[(Ser343Alafs*6)];[Glu442Gly]** | | | | | | | | | | | | | | | | | | | | | | | |
| SOR0081 | M | 4 | -2 | -1 | NA | NA | 7.45 | NA | 129 | 2.3 | 86 | 0.27 | 2.2 | 10.4 | NA | NA | 0.24 | 23 | NA | NA | 0.07 | NA | + |
| **p.[Arg595*];[Arg595*]** | | | | | | | | | | | | | | | | | | | | | | | |
| SOR0080 | M | 11 | -0.98 | -5 | 40 | NA | 7.5 | 29.8 | 135 | 2.1 | 85 | 0.89 | 2.1 | 10.7 | NA | 33 | 1.39 | 38.4 | 1.05 | 13.9 | 0.04 | 0.8 | - |
| **p.[(Leu252fs)];[(Leu252fs)]** | | | | | | | | | | | | | | | | | | | | | | | |
| SOR0090 | M | 0.5 | -5 | -2 | 39 | NA | 7.74 | 60 | 123 | 1.6 | 89 | 0.3 | 1.4 | 9.2 | 19.7 | 30 | 3.75 | 159 | 15 | NA | 3 | NA | - |
| **p.[0];[0]** | | | | | | | | | | | | | | | | | | | | | | | |
| SOR0097 | F | 0.1 | -1 | 1 | 41 | NA | 7.45 | 30.9 | 121 | 3.4 | 67 | 0.35 | 1.4 | 11.6 | NA | NA | NA | 157 | 10.26 | 13 | 4.4 | 7.6 | - |

Abbreviations: SD, standard deviation; Dx, diagnostic; NA, not available; SDS, standard deviation score in comparison with an age- and sex-matched reference population; PH, polyhydramnios; P, plasmatic; HCO3-, bicarbonate; Creat, creatinine; Aldost, aldosterone; FE, fractional excretion; TTKG, transtubular potassium gradient; U, urinary; Ca/Cr, calcium/creatinine ratio; NC, nephrocalcinosis.

*Patients included in our previous manuscript [9].
